# Comprehensive Analysis of NAFLD and the Therapeutic Target Identified

**DOI:** 10.3389/fcell.2021.704704

**Published:** 2021-09-20

**Authors:** Weiheng Wen, Peili Wu, Yugang Zhang, Zijian Chen, Jia Sun, Hong Chen

**Affiliations:** ^1^Department of Endocrinology, Zhujiang Hospital, Southern Medical University, Guangzhou, China; ^2^Department of Endocrinology and Metabolism, Nanfang Hospital, Southern Medical University, Guangzhou, China

**Keywords:** NAFLD, metabolic pathway, immune infiltration, prediction model, therapeutic target, integrated analysis

## Abstract

**Objective:** Non-alcoholic fatty liver disease (NAFLD) is a serious health threat worldwide. The aim of this study was to comprehensively describe the metabolic and immunologic characteristics of NAFLD, and to explore potential therapeutic drug targets for NAFLD.

**Methods:** Six NAFLD datasets were downloaded from the Gene Expression Omnibus (GEO) database, including GSE48452, GSE63067, GSE66676, GSE89632, GSE24807, and GSE37031. The datasets we then used to identify and analyze genes that were differentially expressed in samples from patients with NAFLD and normal subjects, followed by analysis of the metabolic and immunologic characteristics of patients with NAFLD. We also identified potential therapeutic drugs for NAFLD using the Connectivity Map (CMAP) database. Moreover, we constructed a prediction model using minimum depth random forest analysis and screened for potential therapeutic targets. Finally, therapeutic targets were verified in a fatty liver model stimulated by palmitic acid (PA).

**Results:** A total of 1,358 differentially expressed genes (DEGs) were obtained, which were mainly enriched in carbohydrate metabolism, lipid metabolism, and other metabolic pathways. Immune infiltration analysis showed that memory B cells, regulatory T cells and M1 macrophage were significantly up-regulated, while T cells follicular helper were down regulated in NAFLD. These may provide a reference for the immune-metabolism interaction in the pathogenesis of NAFLD. Digoxin and helveticoside were identified as potential therapeutic drugs for NAFLD via the CMAP database. In addition, a five-gene prediction model based on minimum depth random forest analysis was constructed, and the receiver operating characteristic (ROC) curves of both training and validation set reached 1. The five candidate therapeutic targets were ENO3, CXCL10, INHBE, LRRC31, and OPTN. Moreover, the efficiency of hepatocyte adipogenesis decreased after OPTN knockout, confirming the potential use of OPTN as a new therapeutic target for NAFLD.

**Conclusion:** This study provides a deeper insight into the molecular pathogenesis of NAFLD. We used five key genes to construct a diagnostic model with a strong predictive effect. Therefore, these five key genes may play an important role in the diagnosis and treatment of NAFLD, particularly those with increased OPTN expression.

## Introduction

Non-alcoholic fatty liver disease (NAFLD) is one of the most common liver diseases in the world (Chen and Yeh, 2021). NAFLD refers to liver disease without secondary causes, such as drugs, excessive drinking, or genetic diseases, and includes simple fatty liver disease, non-alcoholic steatohepatitis (NASH), and cirrhosis with NASH (Chalasani et al., 2018). The prevalence of NAFLD is increasing worldwide. A recent meta-analysis estimated that the global prevalence of NAFLD is about 25% (Younossi et al., 2016). Another study found that the prevalence of NAFLD in the Asian population was 27% (Younossi et al., 2016). Because of its high prevalence, NAFLD is now the fastest growing cause of liver related mortality worldwide.

Long-term studies have shown that patients with NAFLD have higher overall mortality and liver-specific mortality than that of the general population (Ong et al., 2008). The increase in prevalence of NAFLD will likely be accompanied by a large number of patients with liver cirrhosis and end-stage liver disease requiring liver transplantation (Wong et al., 2015; Goldberg et al., 2017), as well as an increased prevalence of hepatocellular carcinoma (HCC) (Mittal et al., 2016). One study reported that heart-related death is one of the main causes of mortality in NAFLD patients (Ekstedt et al., 2015). These patients are often obese, a comorbidity that is often accompanied by insulin resistance, dyslipidemia, hypertriglyceridemia, and hypertension, which are all risk factors for cardiovascular disease (CVD) (Vernon et al., 2011; Li et al., 2014). Accumulating evidence has shown that the prevalence of NAFLD in patients with metabolic syndrome is relatively high (Liangpunsakul and Chalasani, 2005; Chan et al., 2013). For example, obesity is the cause of a large proportion of NAFLD cases. Recently, experts have reached a consensus that the term “NAFLD” does not reflect the current knowledge, and thus the term metabolic (dysfunction)-related fatty liver “MAFLD” is a more appropriate term (Eslam et al., 2020). Therefore, understanding the role of metabolic regulation in NAFLD is crucial for the development of targeted therapies.

The use of drug combination as treatment for NAFLD is increasing because clinicians worry that the effectiveness of a single agent is not sufficient (Friedman et al., 2018). However, there is no data from long-term controlled trials to confirm the effectiveness of combination therapy. In addition, though the current clinical investigation of therapies for NAFLD is evolving rapidly due to the emergence of new targets and diagnostic techniques, our understanding of NAFLD remains insufficient (Friedman et al., 2018). Therefore, further elucidation of the molecular pathogenesis of NAFLD with an overarching goal of better NAFLD management is warranted.

To address these issues, we developed diagnostic markers for NAFLD and explored the metabolic status of NAFLD patients as well as potential therapeutic targets. We also developed a random forest (RF) prediction model for estimating the status of patients with NAFLD, which had a better performance for predicting NAFLD. In addition, five therapeutic targets for NAFLD (CXCL10, ENO3, INHBE, LRRC31, and OPTN) and several potential therapeutics, including digoxin, were identified.

## Materials and Methods

### Data Acquisition

We downloaded six transcriptome datasets from the National Center for Biotechnology Information (NCBI) GEO public database: GSE24807 (GPL2895, control = 5, NAFLD = 12), GSE37031 (GPL14877, control = 7, NAFLD = 8), gse48452 (GPL11532, control = 41, NAFLD = 32), GSE63067 (GPL570, control = 7, NAFLD = 11), GSE66676 (GPL6244, control = 34, NAFLD = 33), and GSE89632 (GPL14951, control = 24, NAFLD = 39). A PCA plot was used to illustrate the batch effect among the datasets after normalization. Moreover, the Limma package was used for differential analysis to identify differentially expressed genes (DEGs).

### Immune Infiltration

We used the CIBERSORT algorithm (Chen et al., 2018) to analyze the corrected gene expression data and to assess for the presence of invasive immune cells. Notably, CIBERSORT is an important deconvolution algorithm that uses gene expression data and a predefined immune characteristic matrix to estimate the proportion of 22 human immune cells in a given sample. For each sample, the sum of all estimated immune cell types is equal to 1, which reflects the enrichment degree of immune cell infiltration. Furthermore, we compared the difference in immune cell infiltration between NAFLD patients and normal subjects.

### Functional Enrichment Analysis

R package “clusterprofiler” (Yu et al., 2012) was used to annotate the Gene Ontology (GO) and Kyoto Encyclopedia for Genes and Genomes (KEGG) enrichment function of differential genes, with the goal of comprehensively exploring differential gene expression involved in disease progression. GO and KEGG pathways with *P*-value and *Q*-value less than 0.05 were considered as significant categories. In addition, we utilized gene set variation analysis (GSVA) (Hanzelmann et al., 2013), a non-parametric and unsupervised gene set enrichment method, which can be used to calculate the score of pathways or characteristics related to transcriptomic data, then identify the biological function of samples. The metabolic related gene characteristics in this study were obtained from previous published studies (Desert et al., 2017; Yang et al., 2020b). To evaluate the potential metabolic activity between samples, we used the GSVA R package to obtain the corresponding metabolic pathway scores for each sample.

### Drug Sensitivity Analysis

The Connectivity Map (CMAP) database^1^ contains 6100 instances of 1309 small molecule drugs, with each instance containing gene expression profiles for specific drugs and corresponding treatments. In this study, we used gene expression profiles to predict potential molecular compounds for NAFLD therapy based on the CMAP database. Firstly, we analyzed differential expression between NAFLD and normal tissues. Next, 300 genes with the most significant folding changes (150 up-regulated and 150 down-regulated) were submitted to the CMAP website and used to search for small molecule drugs that may improve NAFLD prognosis. The correlation between drugs and DEGs was represented by scores ranging from –1 to 1. Notably, a negative score represents the gene expression pattern of corresponding interference, which is contrary to the specific expression pattern of the disease, indicating that this interference has a potential therapeutic effect.

### Feature Selection and Model Construction

To estimate NAFLD patients in both cohorts, we developed a prediction model based on random forest (RF) analysis. Firstly, the six GEO datasets (GSE48452, GSE63067, GSE666676, GSE89632, GSE2807, and GSE37031) were combined into a queue, and the batch effect was removed using the combat functions in the SVA package. Next, GSE48452, GSE63067, GSE666676, and GSE89632 were combined into a training cohort and used to build a prediction model, while the external verification of model performance was evaluated using GSE2807 and GSE37031 datasets. Secondly, we analyzed the differences in the expression of control and NAFLD samples, and the information gene was selected as input for the RF model (*P* < 0.05). This backward elimination method was then used to find the best biomarkers for random forest analysis. Specifically, we used out of band (OOB) errors as the minimum criteria to eliminate variables by setting the descent score of each iteration to 0.2, which means that 20% of the genes are removed from the bottom of the gene importance ranking list in each iteration until the OOB error rate reaches its minimum (Yang et al., 2020a). When RF reaches the minimum OOB error rate, a set of genes are selected as the best biomarkers and used to establish the final RF prediction model (Yang et al., 2020a). Finally, the predictive performance of the model in training and validation cohorts was evaluated using receiver operating characteristic (ROC) curves.

### Single-Cell RNA-Seq Analysis

Single-cell RNA-seq analysis of GSE158241 was performed by using Seurat package. Low quality cells were excluded according to the following quality control criteria: (1) genes identified in < 3 cells were excluded; (2) cells with total detection gene < 50 were excluded; and (3) cells with more than 5% mitochondria genes were excluded. PCA analysis was used to identify available dimensions. Subsequently, T-SNE algorithm was applied to perform non-linear dimensional reduction and cluster classification across cells. Afterward, the Celldex software package was used to annotate cell clusters, and further cell subpopulations quantification was performed according to GSVA method. The corresponding genes of cell surface markers used for cell cluster annotation were retrieved from Cellmarker and Panglaodb database, and the detailed information of cell markers was listed in Supplementary Table 1.

### RT-PCR, siRNA Transfection, and NAFLD Model Establishment Using Palmitic Acid

HepG2 human hepatoma cells were purchased from the Chinese Academy of Sciences (Shanghai Institute of cell biology, China). The cells were cultured in DMEM medium (GIBCO) containing 10% fetal bovine serum (GIBCO) and 1% antibody (GIBCO), then incubated in 5% CO_2_ at 37°C. When the cell density reached 60% confluence, the cells were transfected with human OPTN siRNA or a negative control siRNA (RiboBio, Guangzhou, China), using Lipofectamine 3000 (Invitrogen, United States) per manufacturer’s instructions. After 24 h of transfection, the cells were cultured with palmitic acid (PA) (Solarbio, Beijing, China) at the concentration of 0.3 nM, while the vehicle control group were treated with PA-free bovine serum albumin (Solarbio, Beijing, China) at the concentration of 1%(w/v) for 24 h. Next, total RNA was extracted using Trizol (Invitrogen, United States), and cDNA was synthesized by reverse transcription using PrimeScript RT Master Mix (Takara, Japan) per manufacturer’s instructions. A TB green premix ex Taq Kit (Takara, Japan) was used for real-time quantitative PCR (RT-PCR) with gene-specific primers; the sequences of the primers is shown in Supplementary Table 2. Each sample was replicated three times, and the relative abundance of transcripts was normalized using β-actin as a control. Finally, the 2^–ΔΔ*CT*^ method was used to calculate the relative change in gene expression.

### Enzyme-Linked Immunosorbent Assay (ELISA) and Intracellular TG Measurement

HepG2 cells transfected with OPTN siRNA or negative control siRNA were continuously incubated and cultured in medium with 0.3 nM PA for 24 h. Then, culture supernatants from HepG2 were collected and analyzed for detection of IL-6, IL-8, ICAM-1, MCP-1, and TNF-α using ELISA kits (MultiSciences, Hangzhou, China) following the manufacturer’s instructions. Intracellular TG concentrations were normalized to protein concentrations. TG levels were determined using a Triglyceride assay kit (Nanjing Jiancheng, Nanjing, China) and protein concentrations were measured using the Pierce BCA protein quantitative assay kit (Thermo-Fisher Scientific, Massachusetts, America).

### Statistical Analysis

All statistical analyses were performed in R (version 3.6), and all statistical tests were bilateral. *P* < 0.05 was considered to be statistically significant.

## Results

### Differential Gene Expression Analysis in NAFLD

**FIGURE 1:**
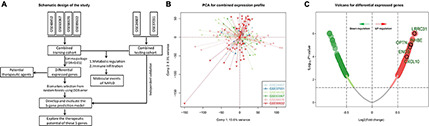
Differentially expressed genes. **(A)** Schematic diagram of the study design. **(B)** Two-dimensional PCA plot of the combined expression profile. **(C)** Volcano of differentially expressed genes. The red dots represent up-regulated genes while the green dots represent down-regulated genes.

The study design is shown in Figure 1A. We downloaded GSE24807, GSE37031, GSE48452, GSE63067, GSE66676, and GSE89632 data sets from the GEO database. A total of 118 normal patients and 135 NAFLD patients were included. Microarray data with batch effect were normalized using scale method, and the PCA plot shows that the batch effect was eliminated among the datasets (Figure 1B). We then analyzed the differentially expressed genes (DEGs) between NAFLD and control samples to identify NAFLD-specific gene expression patterns. The GSE48452, GSE63067, GSE66676, and GSE89632 data sets were used as the training set, followed by differential analysis using the limma package. The screening condition was: adj *P* < 0.05. In total, 1,358 DEGs were obtained, of which, 826 were up-regulated and 532 were down-regulated (Figure 1C and Supplementary Table 3).

### Pathway Analysis of NAFLD Pathogenesis

**FIGURE 2:**
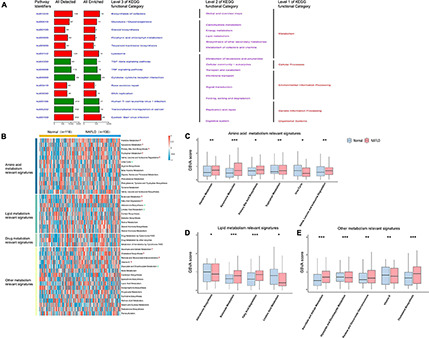
Pathway analysis of NAFLD pathogenesis. **(A)** Pathway analysis of differentially expressed genes. The red bar represents the functional pathway enriched by up-regulated genes, while the green bar represents the functional pathway enriched by down-regulated genes. **(B)** Heatmap of the specific metabolism-associated pathways. **(C)** Boxplot of the signature score for differentially amino acid metabolism-associated pathways. **(D)** Boxplot of the signature score for differentially lipid metabolism-associated pathways. **(E)** Boxplot of the signature score for differentially other metabolism-associated pathways. *p < 0.05, **p < 0.01 and ***p < 0.001.

We annotated the functions of obtained DEGs to further analyze their significance in NAFLD pathogenesis. Up-regulated DEGs were mainly enriched in carbohydrate metabolism, lipid metabolism, and other metabolic processes, while down-regulated genes were mainly enriched in TGF-β signaling, TNF signaling, and cytokine receptor interactions, among other pathways (Figure 2A). Because metabolic dysregulation is involved in NAFLD pathogenesis, we further explored whether the control and NAFLD groups had differing metabolic characteristics. Firstly, we quantified 41 metabolic processes using the GSVA R package, then conducted differential analysis to determine the subclass of specific metabolic characteristics. We found that 11 metabolic characteristics were enhanced in NAFLD, including primary bile acid biosynthesis, and cholesterol biosynthesis (Figures 2B–E).

### Immune Infiltration in NAFLD

**FIGURE 3:**
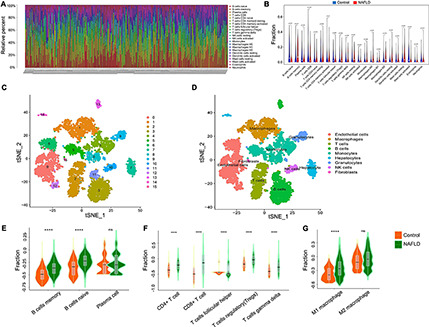
Immune landscape of NAFLD. **(A)** The percentage of 22 types of immune cells in the NAFLD and control groups. **(B)** The difference of immune cells between NAFLD and control group. **(C)** The TSNE algorithm was used for dimensionality reduction and finally 16 cell clusters were successfully classified. **(D)** All 16 clusters of cells were annotated by Celldex package according to the composition of the marker genes. **(E)** Enrichment scores of B cells subpopulations at the single-cell level. **(F)** Enrichment scores of T cells subpopulations at the single-cell level. **(G)** Enrichment scores of Macrophages subpopulations at the single-cell level. ****p < 0.0001.

Immunologic dysregulation is a major driver of NAFLD progression and other metabolic diseases. To determine whether the immune landscape of NAFLD differed from that of healthy subjects, we used CIBERSORT, a non-negative matrix factorization algorithm, to calculate the proportion of different types of immune cells in the tissue according to the LM22 signature matrix. The immune infiltration in each patient’s tissue is shown in Figure 3A. There was a significant difference in the content of immune infiltration between normal liver tissue and that of NAFLD patients (Figure 3B). Moreover, memory B cells, regulatory T cells, resting NK cells, resting dendritic cells, macrophages, and resting mast cells were significantly increased in liver tissues of NAFLD patients, while naïve B cells, plasma cells, T follicular helper cells, activated NK cells, activated mast cells, and neutrophils were significantly decreased. To further verify the above results, we analyzed the scRNA-seq dataset of GSE158241 to infer the difference of immune cell subpopulations between the control group and NAFLD group. By integrated analysis of scRNA-seq and bulk-seq, we can more comprehensively understand the difference of immune infiltration in NAFLD. By using T-SNE algorithm, the cells are successfully divided into 16 clusters (Figure 3C). Subsequently, a total of 9 types of cells are annotated by Celldex package, including T cells, B cells, Macrophages, Monocytes, NK cells, Granulocytes, Endothelial cells, Fibroblasts, and Hepatocytes (Figure 3D). According to the Cellmarker and Panglaodb database, we retrieved the corresponding markers of immune cell subpopulations and used GSVA method to quantified the specific immune cell types. The results showed that memory B cells, regulatory T cells, M1 macrophages cells were significantly increased in NAFLD, while T cells follicular helper significantly decreased (Figures 3E–G), which was consistent with the results of bulk-seq analysis. These findings reveal more accurate characteristic changes of immune infiltration in the development of NAFLD.

### Screening for Potential NAFLD Therapeutic Agents

**FIGURE 4:**
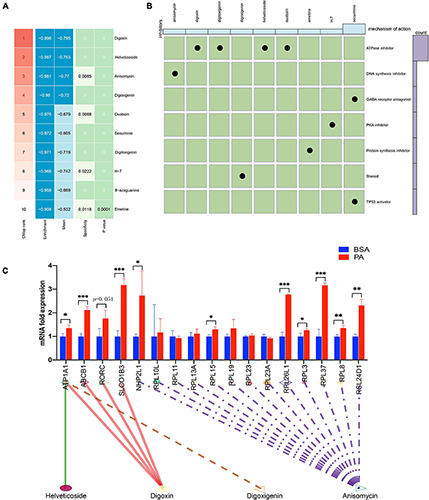
CMap analysis of potential therapeutic drugs for NAFLD. **(A)** Results of CMap analysis for differentially expressed genes. **(B)** Molecular action of potential therapeutic drugs. **(C)** Expression of 17 drugs target genes in HepG2 cell after stimulated by palmitic acid. *p < 0.05, **p < 0.01 and ***p < 0.001.

To screen small molecule drugs targeting NAFLD, we used CMAP analysis to assess gene expression that was increased in NAFLD but was decreased after treatment with a variety of compounds. We uploaded 300 genes with the most significant changes (150 up-regulated genes and 150 down-regulated genes) to the CMAP database, and the top 10 relevant drugs associated with NAFLD treatment were identified (Figure 4A and Supplementary Table 4). Among them, digoxin, helveticoside, anisomycin, and digoxigenin were highly negatively correlated with NAFLD progression, indicating that these compounds may have potential therapeutic effectiveness. Next, we further evaluated the molecular action of these compounds to explore their potential in the treatment of NAFLD. The mechanism of actions (MOA) and drug target of these drugs were analyzed by Clue database^2^ to explore their potential mechanism for treating NAFLD (Figure 4B). the expression of these drugs target in HepG2 cell after palmitic acid stimulation was tested (Figure 4C). The fold-change differences of the expression levels of candidates drug targets between NAFLD and normal status were calculated, and a higher fold change value indicated a greater potential of candidate agent for NAFLD treatment. The tomograms of the top four potential molecular drugs were provided by PubChem, as shown in Supplementary Figure 1.

### Construction of a NAFLD Prediction Model and Target Gene Validation

**FIGURE 5:**
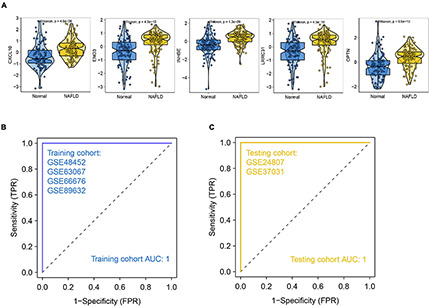
Construction of NAFLD prediction model. **(A)** Expression pattern of the identified hub genes. **(B)** ROC of the training set. **(C)** ROC of the validation set.

**FIGURE 6:**
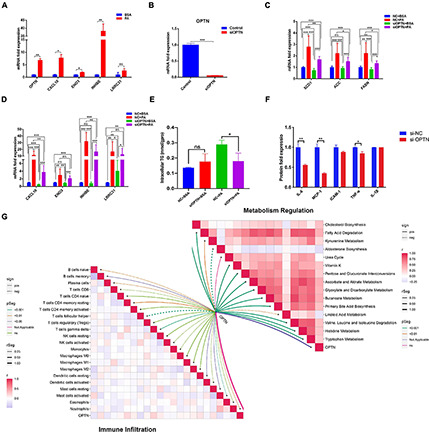
Target gene validation in HepG2 cell. **(A)** Expression of 5 hub genes in HepG2 cell after stimulated by palmitic acid. **(B)** Efficacy of OPTN knockout in HepG2 cell. **(C)** Expression of adipogenesis related genes after OPTN knockdown. **(D)** Expression of other hub genes after OPTN knockdown. **(E)** Effect of OPTN knockdown on the intracellular triglyceride content in HepG2 cell. **(F)** Effect of OPTN knockdown on the release of proinflammatory cytokines in HepG2 cell. **(G)** Metabolic and immune regulation of OPTN. *p < 0.05, **p < 0.01 and ***p < 0.001.

We selected differential genes for further screening through minimum depth random forest analysis. In total, five key genes were screened:CXCL10, ENO3, INHBE, LRRC31, and OPTN. These genes were significantly up-regulated in NAFLD patients compared to controls (Figure 5A). Therefore, we established a prediction model of a five-gene signature based on these key genes. After training the model, we found that the prediction efficiency of ROC in the training set was 1 (Figure 5B). Furthermore, we selected GSE24807 and GSE37031 as external validation sets to explore the predictive stability of the model. The ROC of the external validation set also reached 1 (Figure 5C), suggesting that this model can accurately predict the occurrence of NAFLD. On the other hand, we used a palmitic acid-induced fatty liver model to explore the expression pattern of these five core genes in fatty liver. The results showed that CXCL10, ENO3, INHBE, OPTN were differentially expressed (Figure 6A). As is known to all, NAFLD can develop into liver cancer, which is a high risk factor for liver cancer. According to AJCC staging system, we included data from both TCGA and GEO datasets (GSE36376, GSE84005, and GSE101685) of patients with early stage hepatocellular carcinoma. The results showed that only OPTN was up-regulated in multiple datasets of early hepatocellular carcinoma (Supplementary Figure 2A), which was consistent with the high expression pattern of OPTN in NAFLD, suggesting that OPTN was also a diagnostic marker of early hepatocellular carcinoma and may be involved in the development of NAFLD to hepatocellular carcinoma. In addition, high expression of OPTN is associated with poor prognosis of hepatocellular carcinoma (Supplementary Figure 2B). Therefore, OPTN might have the potential to prevent NAFLD patients from developing hepatocellular carcinoma or a biomarker of early hepatocellular carcinoma. Further study of OPTN in NAFLD has more important clinical significance. OPTN plays an important role in different tissues by regulating endoplasmic reticulum stress (Ali et al., 2019), cell death (Ali et al., 2019), inflammatory responses (Slowicka et al., 2016), and autophagy (Korac et al., 2013; Heo et al., 2015). These pathways are closely related to the occurrence and development of NAFLD. Therefore, we used siRNA to knock down the expression of OPTN in HepG2 cells. We found that the efficiency of adipogenesis and the levels of proinflammatory cytokines (IL-6, MCP-1, TNF-α) were decreased after OPTN knockdown (Figures 6B–F), suggesting that OPTN could delay the adipogenesis in fatty liver. The metabolic and immunologic regulation of OPTN is shown in Figure 6G.

## Discussion

NAFLD is the main cause of fatty liver disease, which is characterized by diffuse alveolar fat within the liver (Chalasani et al., 2018). It is worth noting that NAFLD has become an important cause of chronic liver disease in developed countries due to the globalization of obesity and its related metabolic syndrome. Currently, the identification of NAFLD and its high-risk population is not accurate, and there is lack of sufficient knowledge on its natural history and the key pathogenic factors that stimulate the disease. Therefore, this warrants a further elucidation of NAFLD pathogenesis to provide better treatment strategies. In this study, we used the GEO database to explore diagnostic markers for disease progression and potential NAFLD drug targets. Our results describe a comprehensive immunologic landscape in patients with NAFLD. We also identified several potentially effective drugs, providing an important theoretical basis for the prevention and treatment of NAFLD. In addition, we identified five key genes associated with poor prognosis (CXCL10, ENO3, INHBE, LRRC31, and OPTN) using a minimum depth random forest algorithm and established a prediction model based on these key genes. The predictive efficiency of our model reached 100% in both the training and test sets. These findings may strongly support further comprehensive study of NAFLD precision treatment.

Previous studies have shown that the more components of metabolic disorders coexist in patients with NAFLD, the more likely they are to develop NASH and cirrhosis (Liangpunsakul and Chalasani, 2005; Chan et al., 2013). The role of NAFLD in promoting metabolic syndrome may be associated with the accumulation of liver fat and excess visceral adipose tissues; however, the specific mechanisms are still unclear. In this study, functional enrichment analysis showed that carbohydrate metabolism, lipid metabolism, and other metabolic processes were up-regulated in NAFLD patients. Our ssGSEA results also showed that the up-regulation of differential metabolic pathways was a primary feature of NAFLD, including primary bile acid biosynthesis, cholesterol biosynthesis, and other lipid metabolism-related pathways. Notably, bile acid synthesis is the main pathway for cholesterol and lipid metabolism, whose dysregulation is associated with obesity, diabetes, non-alcoholic fatty liver disease, and other metabolic diseases (Jia et al., 2021). In addition, cholesterol is considered to be the main lipotoxic molecule in the development of NASH, which can promote lipid accumulation and hepatocyte proliferation, thereby leading to the development of NAFLD and even HCC (Ioannou, 2016).

Immunologic dysfunction is one of the main driving factors of NAFLD progression and other obesity related diseases, of which both innate and adaptive immune systems are involved (Van Herck et al., 2019). We used the CIBERSORT algorithm to quantify the immune infiltration of patients to explore whether targeting the regulatory mechanisms of the immune system in NAFLD could be helpful in alleviating intrahepatic inflammatory responses and reducing hepatocyte damage, fibrosis, and even hepatocarcinogenesis. We found that the levels of resting dendric cells, resting NK cells, memory B cells, and regulatory T cells were significantly increased in liver tissues of NAFLD patients. We further performed the scRNA-seq analysis of GSE158241 dataset. The results showed that memory B cells, regulatory T cells, M1 macrophages cells were significantly increased in NAFLD, while T cells follicular helper significantly decreased, which was consistent with the results of above analysis. Some studies have shown an increase number of Treg cells in liver of NAFLD (Bertola et al., 2010; Soderberg et al., 2011), which is consistent with our analysis. The role of Treg cells is related to the regulation of immune system, and the increase of Treg cells subpopulations may indicate the immune disorder leading to liver inflammation in NAFLD. In High fat diet fed mice, the adaptive cell transfer of Treg cells did not cause metabolic disorder rather than aggravated the degree of hepatic steatosis (Van Herck et al., 2020). In addition, hepatic macrophages play an important role in regulating innate immune responses (Kong et al., 2019). Macrophage are a highly heterogeneous immune cell, which shows pleiotropic and coordinated response to the immune environment. The proinflammatory phenotype, M1 macrophage, involved in the pathogenesis of inflammation disorders, including NAFLD (Zhang Y. Y. et al., 2017). LPS or free fatty acid could activate a M1 phenotype and release of inflammation cytokines through TLR4 signal pathway (Koyama and Brenner, 2017). Inhibition of M1 polarization attenuates diet-induced NASH (Zhang Y. Y. et al., 2017). Collectively, our results suggest a more accurate characteristic changes of immune infiltration in the development of NAFLD.

Currently, there are no drugs associated with the treatment of NASH (Friedman et al., 2018). The reuse of known drugs is a feasible drug development strategy. In the past decade, several studies have been conducted with the goal of identifying the genetic features that predict the development of NAFLD. We used the CMAP database to identify potential drugs for NAFLD treatment based on the DEGs identified in our study. The connectivity scores of digoxin, helveticoside, anisomycin, and digoxigenin were relatively low, which suggests that these drugs are highly negatively correlated with NAFLD-specific DEGs. Digoxin and helveticosite are ATPase inhibitors. Recent studies reported that ATPase inhibitors are closely related to fatty oxidation and liver fibrosis (Cortez-Pinto et al., 1999; Orlov et al., 2019), which are the pathological basis of NAFLD development. Digoxin, for example, has been reported to inhibit the secretion of IL-17A, reduce the levels of liver steatosis, and block the infiltration of liver immune cells, thus preventing liver injury. In high-risk patients infected with HBV or HCV, digoxin provide an effective and cheap method of prevention for the development of NASH and HCC (Gomes et al., 2016). In addition, digoxin can effectively maintain the redox homeostasis of hepatocytes, inhibit the activation of the HIF-1α pathway, and protect the liver from inflammation and injury in NASH and ASH (Ouyang et al., 2018). We found that digoxin may have the potential to reverse NAFLD. Anisomycin is a kind of DNA synthesis inhibitor. The results of MOA suggest that it is involved in the regulation of RPL3 expression. Studies have shown that high levels of RPL3 can be found in both polygenic and monogenic obese mouse (Allan et al., 2001). Also, the expression of RPL3 was related to the heat expenditure of brown adipose tissue (Allan et al., 2000; Wesolowski et al., 2003), suggesting that RPL3 was a regulatory factor of energy balance and had the potential to alleviate the progression of NAFLD. Taken together, the reuse of these three drugs is a feasible drug development strategy, which may provides new insights therapeutic options for the treatment of patients with NAFLD.

In the past decade, high-throughput analysis has enabled scientists to restate key events that occur in NAFLD (Gorden et al., 2015; Gjorgjieva et al., 2019). We developed an effective method for predicting NAFLD and identified potential drugs for NAFLD treatment. We obtained a five-gene characteristic using a minimum depth random forest algorithm and identified CXCL10, ENO3, INHBE, LRRC31, and OPTN as key genes in NAFLD progression. In addition, the AUCs of the prediction model were verified in the training and verification sets (both 100%), indicating that these genes are potential diagnostic markers of NAFLD. Previous studies have reported that CXCL10 is a chemotactic ligand derived from hepatocytes (Ibrahim et al., 2016), and initiates an inflammatory cascade through its homologous receptor CXCR3 (Tomita et al., 2016). Another study showed that the expression of CXCL10 is increased in steatohepatitis (Zhang et al., 2014). In contrast, knockout of CXCL10 expression has a protective effect on *in vitro* hepatocyte damage and mouse steatohepatitis (Zhang X. et al., 2017). A previous study reported that mice deficient in CXCL10 or its homologous receptor CXCR3 gene were protected from diet-induced NASH (Tomita et al., 2016). Therefore, CXCL10 may be used as an early indicator or as a target to inhibit inflammatory responses in NAFLD. LRRC31 belongs to the LRRC superfamily, which plays an important role in cell cycle regulation, chromosome stability, apoptosis, and DNA repair (Codd et al., 2010). One study reported that LRRC1 may be an oncogenic gene, and overexpression of LRRC1 accelerated the growth and colony formation of hepatoma cells (Li et al., 2013). In addition, the LRRC31 mRNA was positively correlated with eosinophilia and IL13 and IL5 expression, and was related to cellular immune regulation as well as inflammatory response (D’Mello et al., 2016). These molecular events may be involved in the pathogenesis of NAFLD. Enolase 3 (ENO3), an enzyme that mediates the synthesis of cholesterol esters, is distributed in various tissues, such as the liver, lungs, bone, and heart (Wu et al., 2008). Moreover, it may participate in the regulation of liver lipid transport and energy homeostasis, and is important for the accumulation of cholesterol esters in the liver of obese patients (Stahlberg et al., 1997). A previous study reported that the level of ENO3 was significantly higher in the livers of morbidly obese subjects compared to those who had experienced a great quantity of weight loss (Elam et al., 2009). INHBE belongs to the transforming growth factor-β (TGF-β) family. It is mainly expressed in the liver, where it regulates the growth and differentiation of hepatocytes (Chabicovsky et al., 2003; Vejda et al., 2003). INHBE mRNA has been positively correlated with HOMA-IR and body mass index. Studies have shown that it is a possible insulin resistance-related hepatocyte factor (Sugiyama et al., 2018), and its expression was up-regulated in the liver of obese mice after insulin stimulation (Roberts et al., 1990). In addition, the subunit activin E, encoded by INHBE, can stimulate energy consumption by activating brown and beige adipocytes, suggesting that it may be a target for obesity prevention or treatment (Hashimoto et al., 2018). OPTN is a multifunctional protein involved in signal transduction, vesicular transport, immune response, autophagy and various signaling pathways, including nuclear factor-kappa B (NF-kB) Mutations or deletions of the OPTN gene are associated with severe neurodegenerative diseases, such as amyotrophic lateral sclerosis, and glaucoma, inflammation, and elevated cancer risk. However, the specific role of OPTN in NAFLD is still unknown. In this study, we used PA-stimulated hepatocytes to develop a fatty liver model. Using this model, we found that the expression of CXCL10, ENO3, INHBE, and OPTN was up-regulated in PA-treated hepatocytes. In addition, analysis of immune cell infiltration showed that OPTN was negatively correlated with T cell and B cell infiltration, and positively correlated with dendritic cells and mast cell infiltration. This was consistent with our analysis of immune infiltration differentiation between normal and NAFLD liver tissues. According to our GSVA analysis, several pathways are associated with OPTN, including the NF-kB-mediated TNF-α pathway and the interferon α pathway, both of which regulate immunity and inflammation (Vidal, 2020; Jang et al., 2021). TNF-α has become a major inducer of nutrient and obesity related NAFLD (Perseghin et al., 2003; Shoelson et al., 2006), and the level of serum TNF-α in patients with NAFLD increased significantly (Assuncao et al., 2018). Recent study have reported that TNF-α can promote the activation of NLRC4 inflammasome and thus increase the production of IL-18 and IL-1 β (Chen and Ma, 2019), which can aggravate inflammation and promote disease development. Anti TNF-α agents are also used to reduce the inflammation, necrosis and fibrosis in NAFLD (Li et al., 2011). IFN-α can affect the activation of memory CD8 cells and cytotoxic CD8 cells, as well as the recruitment of macrophages, leading to chronic inflammation characterized by abnormal activation of some pro-inflammatory pathways (Nishimura et al., 2009). Additionally, IFN-α increased hepatic adipogenesis and VLDL secretion, which was obvious in cultured hepatocytes and in patients with IFN-αtherapy (Shinohara et al., 1997; Feingold et al., 1992). Therefore, OPTN might act in the inflammatory mechanisms of NASH as it is involved in TNF and IFNalfa pathways (Quiroga et al., 2019). Our results also suggest that OPTN expression is closely associated with adipogenesis. We speculate that increased OPTN expression might be accompanied by the activation of DCs in adipose tissues and increased adipogenesis. To the best of our knowledge, this is the first study that has associated OPTN expression with liver adipogenesis. Moreover, we found that the expression of lipogenic genes (SCD1, ACC, and FASN) and lipid accumulation in PA-stimulated hepatocytes were reduced in cells with knocked down OPTN expression, further supporting the idea of OPTN as a therapeutic target that can delay the progression of fatty liver disease. In spite of these findings, our research should be cautiously interpreted due to the limitations of our study. Firstly, although this study have performed multiple perspective analyses to investigate the therapeutic potential of these compounds in NAFLD, the more detailed preclinical assay is needed to support conclusion. Secondly, the biological effect of OPTN has been functionally verified in HepG2 cell line, and further animal studies are warranted to validate the role of OPTN in NAFLD.

## Conclusion

In conclusion, we have successfully provided a more in-depth insight into the overall molecular changes that occur during the pathogenesis of NAFLD, including key events such as metabolic landscape modification and abnormal immune infiltration. Our results provided a reference for the immunologic-metabolic crosstalk that may drive NAFLD progression. In addition, we identified several drugs for the treatment of NAFLD along with five potential therapeutic targets (ENO3, CXCL10, INHBE, LRRC31, and OPTN). We also verified the potential of using OPTN as a new therapeutic target for NAFLD. Collectively, these findings will help provide a better understanding of the occurrence and development of NAFLD.

## Data Availability Statement

The datasets presented in this study can be found in online repositories. The names of the repository/repositories and accession number(s) can be found in the article/Supplementary Material.

## Author Contributions

HC and JS: design and review. WW and PW: analysis and interpretation of data, and writing and revision of the manuscript. YZ: writing and revision of the manuscript. ZC: revision of the manuscript. All authors read and approved the final manuscript.

## Conflict of Interest

The authors declare that the research was conducted in the absence of any commercial or financial relationships that could be construed as a potential conflict of interest.

## Publisher’s Note

All claims expressed in this article are solely those of the authors and do not necessarily represent those of their affiliated organizations, or those of the publisher, the editors and the reviewers. Any product that may be evaluated in this article, or claim that may be made by its manufacturer, is not guaranteed or endorsed by the publisher.

## Footnotes

1
https://portals.broadinstitute.org/cmap/

2
https://clue.io/

## Supplementary Material

Supplementary Figure 1 The molecular structures of potential therapeutic drugs. **(A)** Molecular structures of the digoxin. **(B)** Molecular structure of the helveticoside. **(C)** Molecular structure of the anisomycin. **(D)** Molecular structure of the digoxigenin.

Supplementary Figure 2 The role of OPTN in hepatocellular carcinoma. **(A)** The expression pattern of 5 hub genes in early stage hepatocellular carcinoma in TCGA and GEO datasets. **(B)** High expression of OPTN is associated with poor prognosis of hepatocellular carcinoma.

Supplementary Table 1 Immune cell markers.xls.

Supplementary Table 2 Primer sequence.xls.

Supplementary Table 3 Limma results in data.xls.

Supplementary Table 4 The result of CMap.xls.
