# Supplementary figures and images for: Comprehensive Analysis of NAFLD and the Therapeutic Target Identified
Source: Front Cell Dev Biol. 2021 Sep 20;9:704704. doi: 10.3389/fcell.2021.704704 (PMC8488166; doi:10.3389/fcell.2021.704704)

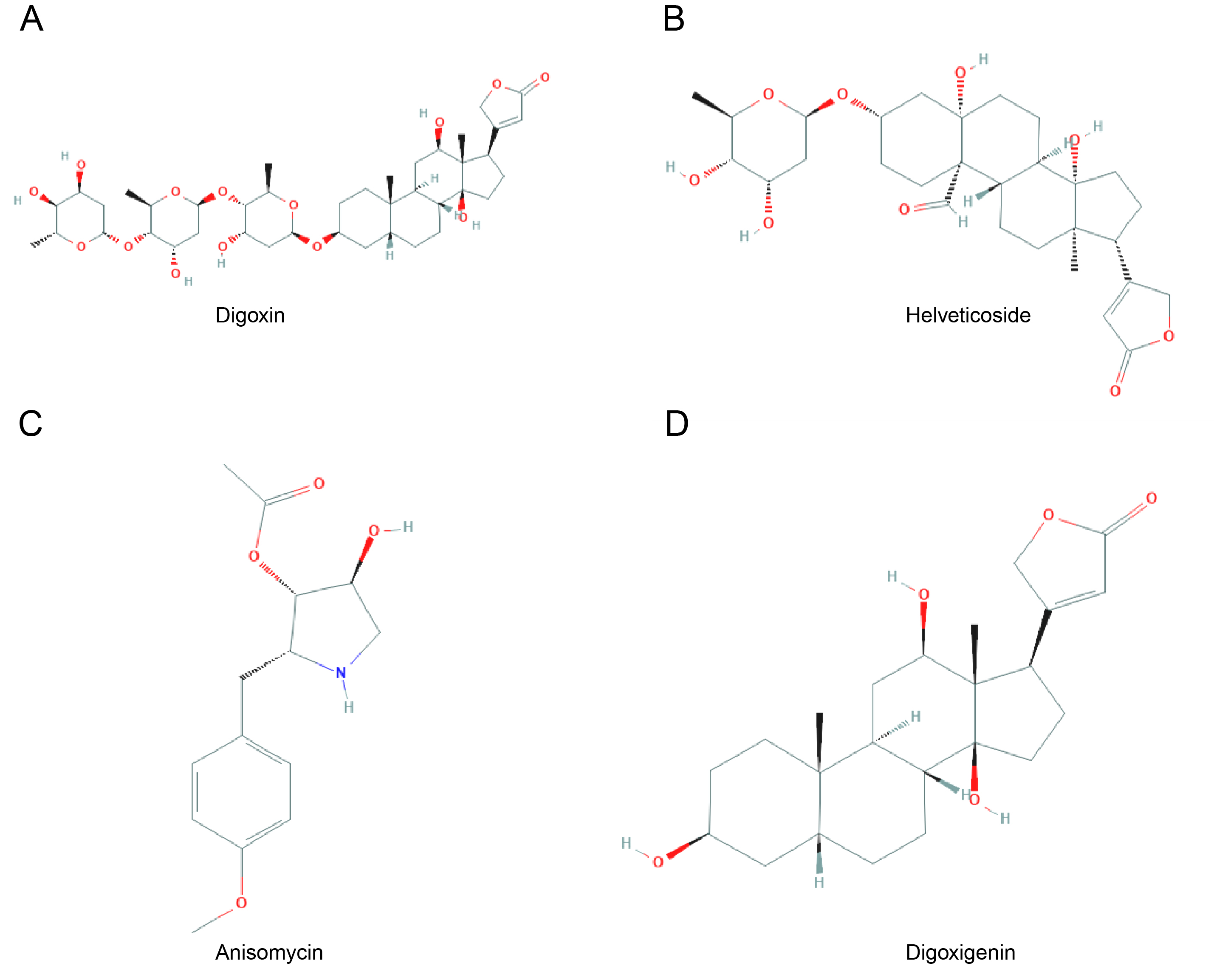

Supplement: Supplementary Figure 1 — The molecular structures of potential therapeutic drugs. (A) Molecular structures of the digoxin. (B) Molecular structure of the helveticoside. (C) Molecular structure of the anisomycin. (D) Molecular structure of the digoxigenin. [file Image_1.JPEG]

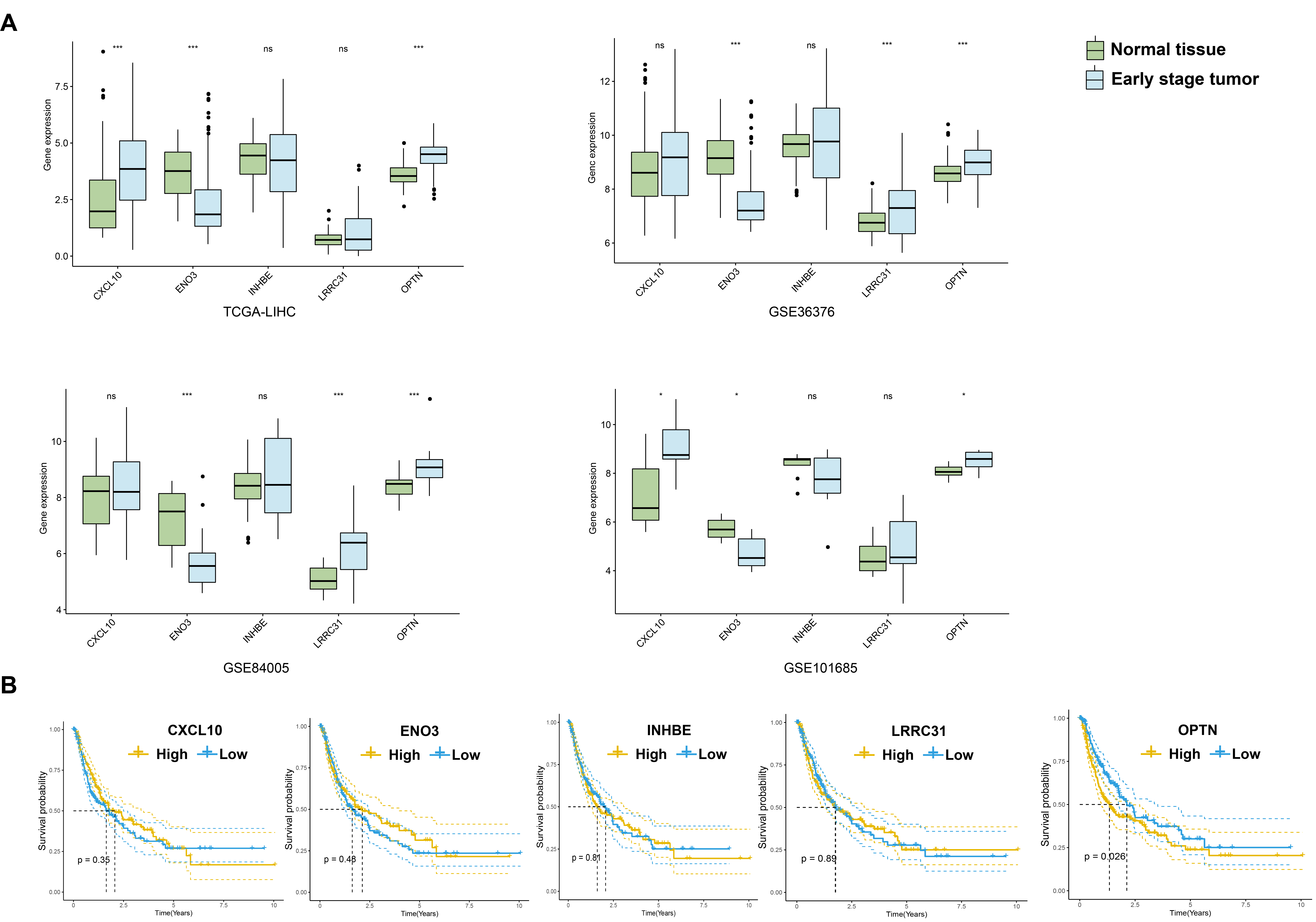

Supplement: Supplementary Figure 2 — The role of OPTN in hepatocellular carcinoma. (A) The expression pattern of 5 hub genes in early stage hepatocellular carcinoma in TCGA and GEO datasets. (B) High expression of OPTN is associated with poor prognosis of hepatocellular carcinoma. [file Image_2.JPEG]
